# Supplementary material for: From sequence to enzyme mechanism using multi-label machine learning
Source: BMC Bioinformatics. 2014 May 19;15:150. doi: 10.1186/1471-2105-15-150 (PMC4229970; doi:10.1186/1471-2105-15-150)
Supplement: Additional file 2 — Java code of ml2db. Additional file ml2db_code.tar.gz contains the Java source code to run the multi-label machine learning experiments and save the results to database. The code’s Javadoc is included. [file 1471-2105-15-150-S2.zip › additional file 2/ml2db/ml2db/doc/test/ml2db/weka/package-summary.html]

test.ml2db.weka


JavaScript is disabled on your browser.


- Overview
- Package
- Class
- Use
- Tree
- Deprecated
- Index
- Help

- Prev Package
- Next Package

- Frames
- No Frames

- All Classes

# Package test.ml2db.weka

- Class Summary

  | Class | Description |
  | --- | --- |
  |  |  |
  | --- | --- |
  | ArffPropsTableTest | Class |
  | ArffTest | Class |
  | AttributeFactoryTest | Class |
  | DataSetCheckerTest | Class |
  | DataSetDbLoaderTest | Class |
  | DataSetGeneratorTest | Class |
  | DataSetManagerTest | Class |
  | DataSetWriterTest | Class |
  | InstancesFillerTest | Class |
  | InstAttValueMapTest |  |
  | WekaTest | Class to test basic weka instance creation |

- Overview
- Package
- Class
- Use
- Tree
- Deprecated
- Index
- Help

- Prev Package
- Next Package

- Frames
- No Frames

- All Classes
